# Attenuation and efficacy of live-attenuated Rift Valley fever virus vaccine candidates in non-human primates

**DOI:** 10.1371/journal.pntd.0006474

**Published:** 2018-05-09

**Authors:** Darci R. Smith, Sara C. Johnston, Ashley Piper, Miriam Botto, Ginger Donnelly, Joshua Shamblin, César G. Albariño, Lisa E. Hensley, Connie Schmaljohn, Stuart T. Nichol, Brian H. Bird

**Affiliations:** 1 United States Army Medical Research Institute of Infectious Diseases (USAMRIID), Fort Detrick, MD, United States of America; 2 Centers for Disease Control and Prevention, Viral Special Pathogens Branch, Atlanta, GA, United States of America; Fundacao Oswaldo Cruz, BRAZIL

## Abstract

Rift Valley fever virus (RVFV) is an important mosquito-borne veterinary and human pathogen that has caused large outbreaks of severe disease throughout Africa and the Arabian Peninsula. Currently, no licensed vaccine or therapeutics exists to treat this potentially deadly disease. The explosive nature of RVFV outbreaks and the severe consequences of its accidental or intentional introduction into RVFV-free areas provide the impetus for the development of novel vaccine candidates for use in both livestock and humans. Rationally designed vaccine candidates using reverse genetics have been used to develop deletion mutants of two known RVFV virulence factors, the NSs and NSm genes. These recombinant viruses were demonstrated to be protective and immunogenic in rats, mice, and sheep, without producing clinical illness in these animals. Here, we expand upon those findings and evaluate the single deletion mutant (ΔNSs rRVFV) and double deletion mutant (ΔNSs-ΔNSm rRVFV) vaccine candidates in the common marmoset (*Callithrix jacchus*), a non-human primate (NHP) model resembling severe human RVF disease. We demonstrate that both the ΔNSs and ΔNSs-ΔNSm rRVFV vaccine candidates were found to be safe and immunogenic in the current study. The vaccinated animals received a single dose of vaccine that led to the development of a robust antibody response. No vaccine-induced adverse reactions, signs of clinical illness or infectious virus were detected in the vaccinated marmosets. All vaccinated animals that were subsequently challenged with RVFV were protected against viremia and liver disease. In summary, our results provide the basis for further development of the ΔNSs and ΔNSs-ΔNSm rRVFV as safe and effective human RVFV vaccines for this significant public health threat.

## Author summary

Rift Valley fever (RVF) is an important neglected tropical disease that has caused severe epidemics and epizootics throughout Africa and the Arabian Peninsula. Severe outbreaks have involved tens of thousands of both human and livestock cases for which no effective, commercially available human vaccines are available. Vaccine candidates have been developed based on the complete deletion of two known RVF virus virulence factors, the NSs and NSm genes. These vaccines were previously demonstrated to be protective in rats, mice, and sheep. In this study, we expand upon those results and evaluate the vaccine candidates in a non-human primate model for RVF. The animals received a single dose of vaccine that led to the development of a robust immune response. No vaccine-induced adverse reactions, signs of clinical illness or infectious virus were detected in the vaccinated animals. All vaccinated animals that were subsequently challenged with RVF virus were protected against viremia and liver disease. These results demonstrate that the vaccines are safe and effective in non-human primates, which provides the impetus for further development of these candidates for use in humans.

## Introduction

Rift Valley fever virus (RVFV; family *Phenuiviridae*, genus *Phlebovirus*) was first isolated in 1930 in East Africa [1] and has since caused severe epidemics and epizootics that affects ruminants and humans throughout Africa and the Arabian peninsula [2, 3]. Human infections result from infected mosquitoes (*Culex*, *Mansonia* and *Anopheles* mosquitoes appear to be the principal vectors for humans [4]) or by contact with tissues, blood, or fluids from infected animals. Human cases are typically self-limiting febrile illnesses and recovery occurs without major consequences. Severe cases, which affect around 1–2% of infected individuals, are characterized by acute-onset liver disease, delayed-onset encephalitis, retinitis, blindness, or a hemorrhagic syndrome, with a case fatality ratio of 10–20% in hospitalized individuals [5–7]. Human cases have been reported in much of Africa, Saudi Arabia, and Yemen [8]. The spread of RVFV into other geographic regions is a major global concern. The productive experimental infection of mosquitoes from multiple distinct geographical regions (including the most widespread vector, *Culex pipiens*) reinforces the feasibility of accidental or intentional import of RVFV from endemic regions with subsequent maintenance in nascent vector and host populations [9–12]. The emergence of RVF into new locations has important implications for human health and livestock industries leading to its identification as a notifiable disease by the World Organization for Animal Health [13] and the World Health Organization as a high priority pathogen requiring attention [14]. Furthermore, due to concerns regarding its use as a potential biological weapon, RVFV has been identified as a Category A, high-priority select agent, by the National Institute for Allergy and Infectious Diseases (NIAID), the Centers for Disease Control and Prevention (CDC), and the United States (U.S.) Department of Agriculture (USDA).

Clearly, RVFV is an important threat to human and animal health for which no specific treatment currently exists. Several RVFV vaccines have been developed [8], but currently none of these candidates has been approved for human use. The formalin-inactivated vaccine TSI GSD 200 was developed by the U.S. Army to protect at-risk laboratory workers against occupational exposure. However, a significant drawback of this vaccine is it requires three inoculations over a 4-week period, a mandatory boost at 6 months, and many recipients require periodic boosters thereafter [15–17]. To overcome these limitations, several live-attenuated vaccines were developed such as the Smithburn and MP-12 vaccines. The Smithburn vaccine has been used in Africa, but has been associated with teratogenesis and abortions in livestock and retains neurovirulence in non-human primates [18, 19]. The MP-12 vaccine was developed by the U.S. Department of Defense [20] and has undergone Phase 1 and 2 clinical trials [21, 22]. Additionally, the MP-12 vaccine is conditionally licensed for veterinary use in the U.S. despite a report that the vaccine may cause teratogenesis or abortions in pregnant ruminants [23]. Furthermore, the MP-12 vaccine lacks a marker for the differentiation of vaccinated from infected animals (DIVA).To overcome some of the limitations of previous live-attenuated vaccines, Bird et al. [24, 25] used reverse genetics to develop a recombinant RVFV, ΔNSs-ΔNSm rRVFV, which contains complete gene deletions of the 2 known RVFV virulence factors, the NSs and NSm genes [24, 26–28]. RVFV has a tripartite negative-stranded RNA genome designated Small (S), Medium (M), and Large (L). The S-segment encodes, in an ambisense fashion, the virus nucleoprotein (NP) in the genomic (negative-sense [–]) orientation and the nonstructural (NSs) protein in the antigenomic (positive-sense [+]) orientation. The M-segment contains at least four nested proteins in a single open reading frame (ORF): the two structural glycoproteins, Gn and Gc, and two nonstructural proteins, the 14-kDa NSm and the 78-kDa NSm-Gn fusion protein. The L-segment encodes the viral RNA-dependent RNA polymerase. The NSs protein is involved in several functions in infected cells such as inhibition of IFN-β, degradation of protein kinase R (PKR), suppression of host transcription, and interactions with host cell chromosome structures [29–34]. The NSm gene is not as well characterized, but has been implicated to be involved in suppression of virus-induced apoptosis [28]. NSs and NSm are not required in cell culture for efficient virus replication, assembly, or maturation [28, 34–36].

The rRVFV vaccine candidates containing the insertion of the enhanced green fluorescent protein and the precise deletion of the NSs gene alone (ΔNSs:GFP rRVFV) or the NSs/NSm genes in combination (ΔNSs:GFP-ΔNSm rRVFV) were described by Bird et al. to be highly attenuated, immunogenic, and efficacious in the rat lethal disease model [24]. Furthermore, the ΔNSs-ΔNSm rRVFV vaccine was demonstrated to be protective and immunogenic in sheep without producing clinical illness in these animals [24, 25]. Importantly, the vaccine was nonteratogenic in pregnant sheep, which is critical to indicate the safety needed for a veterinary vaccine in a natural RVFV host. While the demonstrated safety and efficacy in a natural target species helps to facilitate the acceptance of a vaccine for human use, it is important to determine immunogenicity and efficacy in a species more closely resembling humans. Thus, we completed an evaluation of the single deletion mutant (ΔNSs rRVFV) and double deletion mutant (ΔNSs-ΔNSm rRVFV) vaccine candidates in non-human primates (NHP).

Rhesus macaques historically have been used to evaluate potential vaccines and therapeutics for RVFV [37]. We previously described the development of a NHP model for RVF using the common marmoset (*Callithrix jacchus*). Marmosets were more susceptible to RVFV than rhesus macaques and experienced higher rates of morbidity, mortality, and viremia and marked aberrations in hematological and chemistry values. Depending on the route of exposure, these animals exhibited acute-onset hepatitis, delayed-onset encephalitis, and hemorrhagic disease, which are dominant features of severe human RVF [38]. An additional study compared the susceptibility of rhesus macaques, cynomolgus macaques, African green monkeys, and marmosets exposed to RVFV by aerosol [39, 40]. Cynomolgus and rhesus macaques developed mild fevers, but no other clinical signs were observed and all the monkeys survived. In contrast, African green monkeys and marmosets were found to be highly susceptible to aerosol infection where the majority of animals developed fatal encephalitis [39, 40]. Collectively, these studies highlight the utility of the marmoset model of RVF to evaluate potential medical countermeasures because of their ability to mimic different features of severe human disease.

Here, we demonstrate that both the single deletion mutant (ΔNSs rRVFV) and double deletion mutant (ΔNSs-ΔNSm rRVFV) vaccine candidates were found to be generally safe and immunogenic in a marmoset model of RVF. The vaccinated marmosets exhibited no signs of clinical illness post-vaccination and post-challenge and developed strong neutralizing antibody titers. Our results provide the basis for further development of the ΔNSs and ΔNSs-ΔNSm rRVFV as safe and effective human RVFV vaccines for this important public health threat.

## Methods

### Ethics statement

This work was supported by an approved USAMRIID IACUC animal research protocol (AP-10-066). Research was conducted under an IACUC approved protocol in compliance with the Animal Welfare Act, PHS Policy, and other Federal statutes and regulations relating to animals and experiments involving animals. The facility where this research was conducted is accredited by the Association for Assessment and Accreditation of Laboratory Animal Care, International and adheres to principles stated in the Guide for the Care and Use of Laboratory Animals, National Research Council, 2011. Approved USAMRIID animal research protocols undergo an annual review every year. Animals are cared for by a large staff of highly qualified veterinarians, veterinary technicians, and animal caretakers. All personnel caring for and working with animals at USAMRIID have substantial training to ensure only the highest quality animal care and use. Humane endpoints were used during all studies and marmosets were humanely euthanized when moribund according to an endpoint score sheet.

### Viral strain, animals and study design

Construction of the rRVFV has been previously described [24, 26, 35] and a schematic of the rRVFV reverse genetics rescue system and locations of the NSs and NSm gene deletions were described by Bird et al. [25]. Recombinant viral strain ZH501 was rescued as previously described [26] and the exact complete genome sequence confirmed by techniques described by Bird et al. [41]. Strain ZH501 was originally isolated from a fatal human case during the 1977 epidemic in Egypt and the complete genome sequences of the S, M, and L segments used in this work can be found under GenBank accession numbers DQ380149, DQ380200, and DQ375406, respectively.

Seventeen healthy adult marmosets (*Callithrix jacchus*), 1 to 3 years old and ranging in weight from 257 to 398g were obtained from World Wide Primates (S1 Table). None of these primates was exposed to any infectious pathogens in previous studies and all primates were determined to be RVFV naïve by plaque reduction neutralization test (PRNT; methods below) before the initiation of the study.

For the study design, two groups of six RVFV seronegative marmosets were inoculated subcutaneously with 5 log_10_ PFU of the ΔNSs or ΔNSs/ΔNSm vaccine candidate and one group of five RVFV seronegative marmosets served as the sham-vaccinated controls. This vaccination dose was determined based on our previous studies in rodents and sheep [24, 25]. All animals were monitored for weight loss/survival/clinical signs of infection and blood samples were collected from anesthetized animals on days -3, 0, 2, 4, 7 and once a week thereafter for virological, hematological, immunological, and chemical analyses. Body temperature was monitored rectally. Following vaccination, the marmosets were challenged subcutaneously with 6.4 log_10_ PFU of the virulent strain (ZH501) 35 days post-vaccination. This dose was chosen based on our previous model development study [38]. Following challenge, all animals were monitored for weight loss/survival and blood samples were collected on days 0, 2, 4, 7 and once a week thereafter for virological, hematological, immunological, and chemical analyses. The study endpoint was euthanasia when moribund or 28 days post-challenge (lethal strain)/day 63 of study. Marmosets were euthanized by being deeply anesthetized by injection of Telazol followed by exsanguination. Following euthanasia, a full necropsy was performed for collection of tissues.

### Hematology, blood chemistries and virological assays

Whole blood was added to an EDTA tube (Sarstedt, Numbrecht, Germany) for complete blood count (CBC) determination using a Hemavet instrument (Drew Scientific, Dallas, TX) according to manufacturer’s instructions. Clinical chemistry analyses were performed by addition of whole blood to a lithium heparin tube (Sarstedt) using the comprehensive diagnostic panel analyzed on a Vetscan instrument (Abaxis, Union City, CA) according to manufacturer’s instructions. Normal ranges in the chemistry and hematology results of healthy marmosets [42] were used as reference values. The plasma was then collected for viral titer determination by quantitative RT-PCR (qRT-PCR) as previously described where the limit of detection (LOD) was 0.1 PFU [24, 43] or by standard plaque assay as previously described [44]. A minimum amount of plasma remained (less than 100 μL) so the sample was diluted 10-fold prior to the plaque assay yielding a LOD of 100 PFU. For the qRT-PCR assay, a standard curve was generated using serial dilutions of the challenge virus in triplicate on the LightCycler 480 (Roche Diagnostics, Inc., Indianapolis, IN). The virus titers were calculated using the standard curve and the LightCycler 480 software, and the final PFU equivalents/mL (PFUe/mL) calculations were determined based on the sample input volume and the upfront sample dilutions. At the time of necropsy, the following tissues were collected for viral titer determination: liver, cerebrum, spleen, kidney, lung, heart, adrenal gland, inguinal lymph node, axillary lymph node, mesenteric lymph node, duodenum, jejunum, ileum, ovaries/testis, skeletal muscle, bone marrow, and retina. Tissues were collected, weighed, and homogenized in EMEM containing 5% fetal bovine serum and gentamicin. Tissues were homogenized using the Qiagen Mixer Mill 300 (Retsch, Newtown, PA) then centrifuged at 9,000 x g for 10 min and the supernatant stored at -70°C until further evaluation. Tissues collected at the study endpoint were homogenized according to the methods above and a 1:10 dilution of the supernatant added to 24-well plate of Vero cells in duplicate in a volume of 100 μL for each well. Plates were incubated for 1 h at 37°C with rocking every 15 min. After the incubation, 0.5 mL of EMEM was added to each well and incubated for 4 days to monitor for cytopathic effects (CPE).

### Histopathology

Full necropsies and histological examination were performed by a board-certified veterinary pathologist. The following tissues were collected during necropsy: Axillary, inguinal, submandibular, mesenteric and tracheobronchial lymph node; submandibular salivary gland; haired skin; brachial plexus; sciatic nerve; skeletal muscle; bone marrow (femur); eyes; brain; pituitary gland; spleen; adrenal gland; kidney; liver; stomach; duodenum; pancreas; jejunum; ileum; cecum; colon; testis/ovary; prostate gland/uterus; urinary bladder; tongue; tonsil; trachea; esophagus; thyroid gland; lung; thymus; and heart. All collected tissues were immersion-fixed in 10% neutral buffered formalin for at least 21 days. The tissues were trimmed and processed according to standard protocol [45]. Histology sections were cut at 5 to 6 μm on a rotary microtome, mounted on glass slides, and stained with hemotoxylin and eosin. For immunohistochemical analysis, serial sections of tissue were cut and stained for RVF antigen using a mouse monoclonal antibody (4D4) against the glycoprotein Gn [46, 47] and an immunoperoxidase assay system (EnVision; DAKO). Normal hepatic tissue served as the negative control; the positive control tissue was liver from a known RVF-positive animal. Normal mouse IgG was used as the negative serum control for the control slides. For the immunohistochemistry study, the unstained tissue sections were deparaffinized, rehydrated, subjected to methanol-hydrogen peroxide block, rinsed, and pretreated with Tris/EDTA buffer at 97°C for 30 min. A serum-free protein block (DAKO) plus 5% normal goat serum was applied for 30 min. The primary antibody was then applied to the tissue at a dilution of 1:100 and incubated at room temperature overnight. The tissue sections were rinsed and then exposed to the EnVision horseradish peroxidase labeled polymer for 30 min at room temperature. All sections were exposed to DAB permanent chromogen for about 5 min, rinsed, counter-stained with hematoxylin, dehydrated, and applied a coverslip with Permount.

### Serology

Anti-RVFV total IgG ELISA was performed essentially as described previously [24], with the following modifications necessary for NHP specimens. BHK cell lysate was used rather than Vero E6 cells and the secondary goat anti-monkey IgG horseradish peroxidase-conjugated antibody (KPL, 074-11-021), which was raised against rhesus macaques and most likely contributed to low adjusted sum optical density (OD) values. Neutralizing antibodies were assayed in plasma for marmosets with a 50% PRNT as previously described [48].

### Statistical analysis

Repeated measures ANOVA was used to compare chemistry, viremia, weight, temperature, and antibody response over time and between groups. All analyses were conducted using GraphPad Prism 7.00 (La Jolla, CA).

## Results

**Fig 1:**
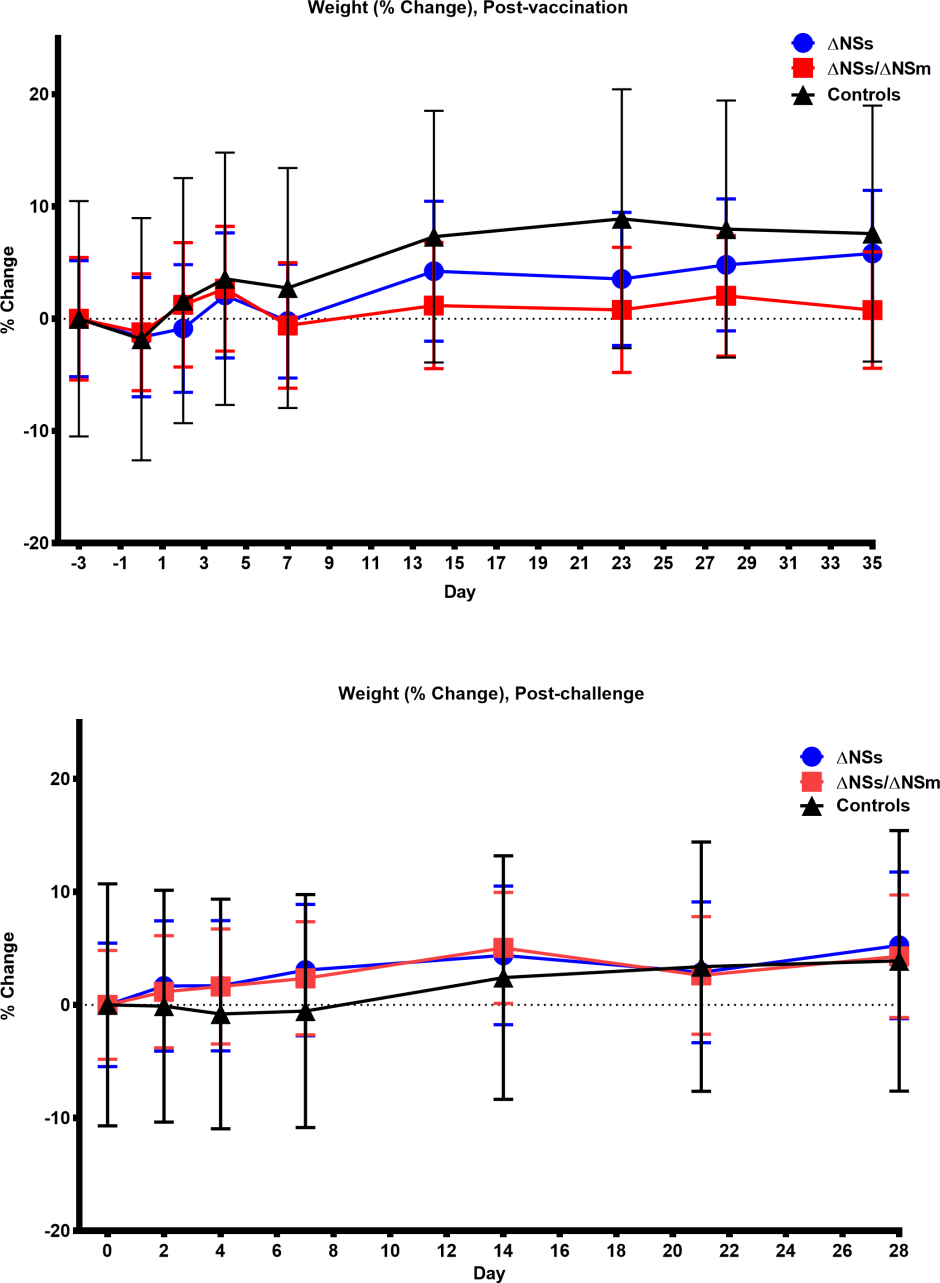
**Weights of marmosets post-vaccination (top) and post-challenge (bottom).** Percent change in baseline of weights of marmosets post-vaccination (top) with rZH501-ΔNSs (n = 6), rZH501-ΔNSs-ΔNSm (n = 6), or sham inoculated controls (n = 5) and post-challenge (bottom) with 6 log_10_ PFU of the virulent strain ZH501. The symbols represent the mean value and the error bars represent the standard error of the mean.

**Fig 2:**
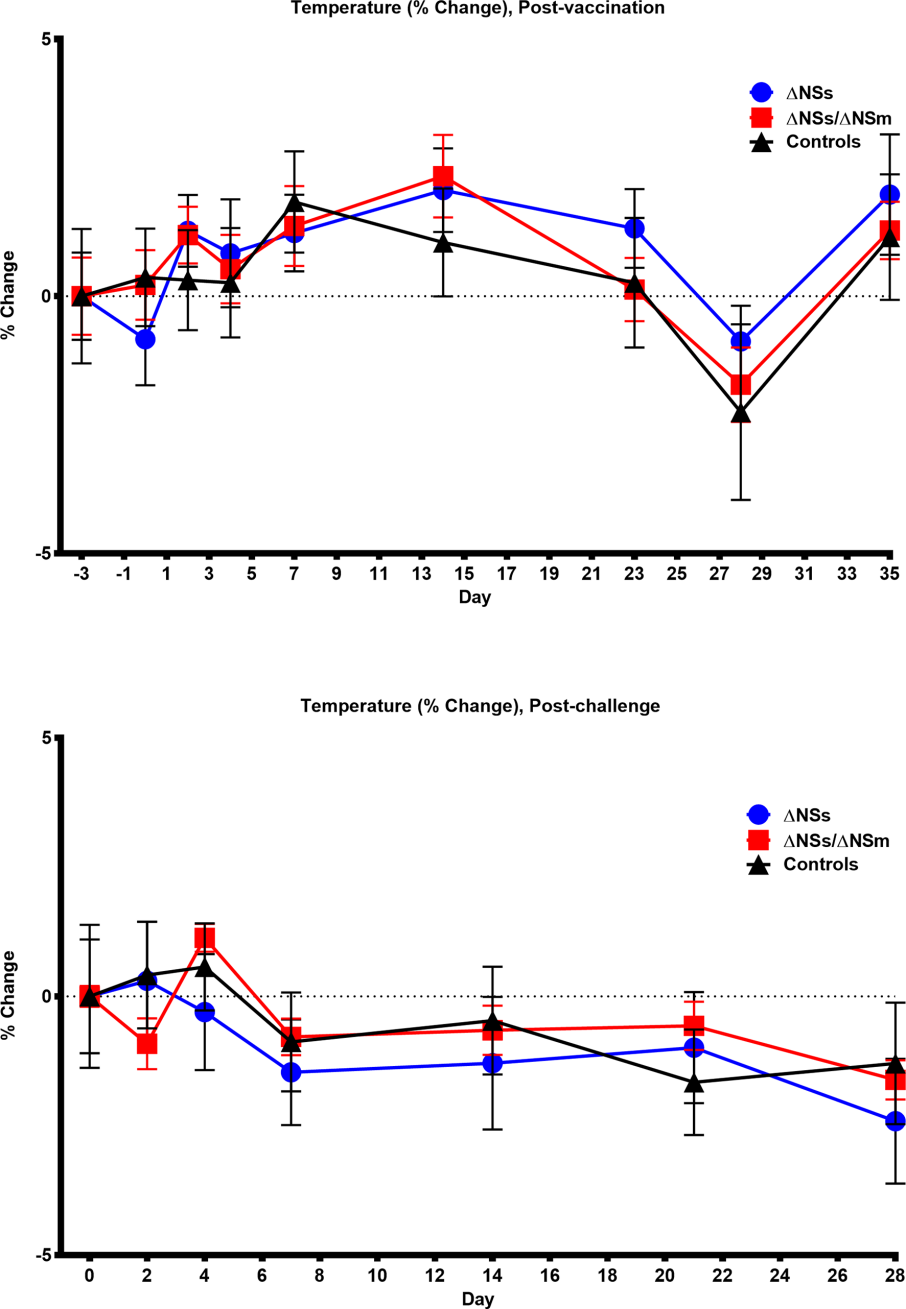
**Temperatures of marmosets post-vaccination (top) and post-challenge (bottom).** Percent change in baseline of the temperature of marmosets post-vaccination (top) with rZH501-ΔNSs (n = 6), rZH501-ΔNSs-ΔNSm (n = 6), or sham inoculated controls (n = 5) and post-challenge (bottom) with 6 log_10_ PFU of the virulent strain ZH501. The symbols represent the mean value and the error bars represent the standard error of the mean.

Marmosets (n = 6/group) were inoculated subcutaneously with 5 log_10_ PFU of the ΔNSs or ΔNSs/NSm rRVFV while five marmosets served as the sham-vaccinated controls. All animals were monitored for weight loss, survival, and clinical signs of infection and blood samples were collected for virological, hematological, immunological, and chemical analyses. All of the vaccinated and sham-vaccinated marmosets survived and no animals exhibited clinical signs of illness, experienced significant weight loss (Fig 1, S1 Fig) or temperature changes (Fig 2, S2 Fig) post-vaccination or post-challenge. Additionally, no animals experienced an adverse reaction at the site of vaccination.

**Fig 3:**
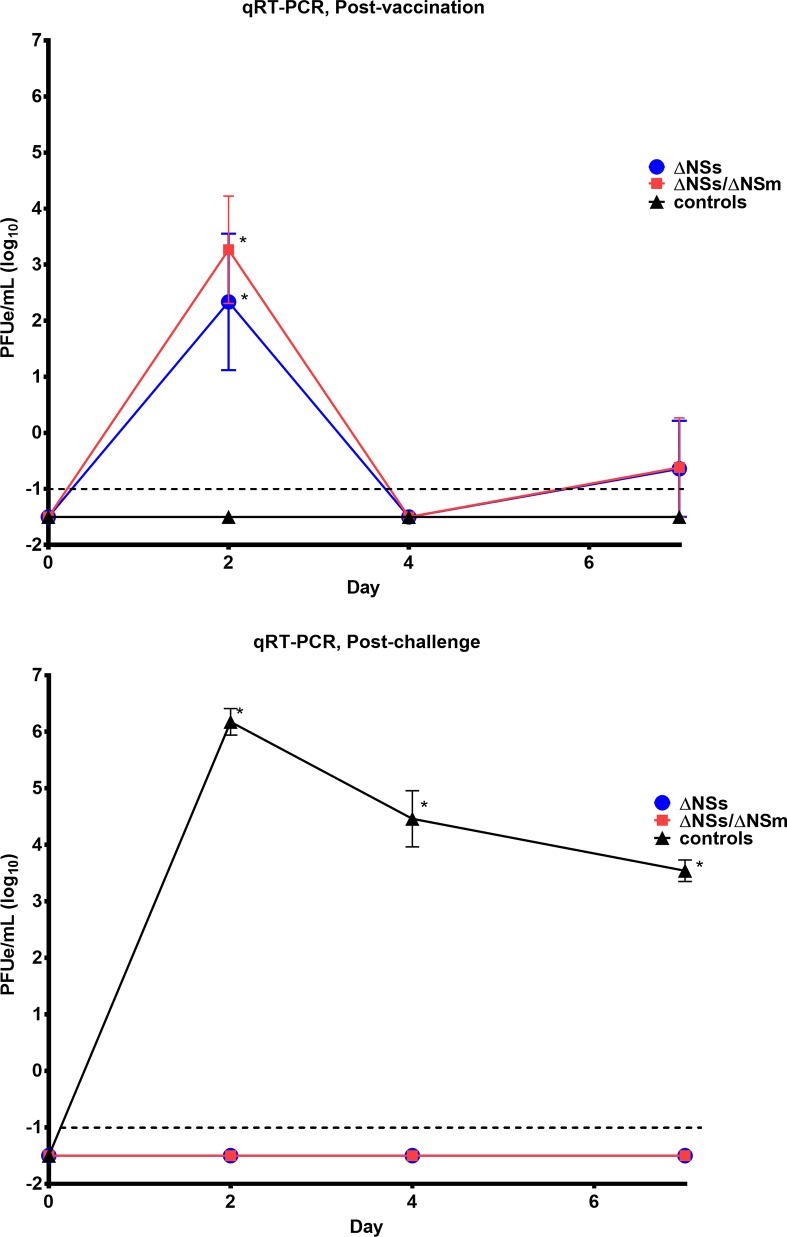
**Viremia determined by qRT-PCR in marmosets post-vaccination (top) and post-challenge (bottom).** RNA detected by qRT-PCR in marmosets post-vaccination (top) with rZH501-ΔNSs (n = 6), rZH501-ΔNSs-ΔNSm (n = 6), or sham inoculated controls (n = 5) and post-challenge (bottom) with 6 log_10_ PFU of the virulent strain ZH501. The symbols represent the mean value and the error bars represent the standard error of the mean. The dashed line represents the assay LOD. PFUe, plaque-forming unit equivalent. Asterisk (*) indicates significantly different values when comparing post-vaccination (top) ΔNSs vs. controls p = 0.0002 and ΔNSs-ΔNSm vs. controls p<0.0001; Post-challenge (bottom) ΔNSs vs. controls and ΔNSs-ΔNSm vs. controls p<0.0001.

Viral RNA was detected on day 2 post-vaccination by qRT-PCR in 4/6 animals that received ΔNSs rRVFV and 5/6 animals that received ΔNSs-ΔNSm rRVFV (Fig 3, S3 Fig). Viral RNA was not detected in any of the animals on day 4 post-vaccination, but was detected for two animals (one animal that received ΔNSs rRVFV and one animal that received ΔNSs-ΔNSm rRVFV) on day 7 post-vaccination. These same samples were evaluated for infectious virus by standard plaque assay and no virus was detected. However, a minimum amount of sample volume was left and had to be diluted ten-fold prior to completion of the plaque assay thus reducing the sensitivity to detect infectious virus where the LOD was 100 PFU. Regardless, these results suggest that little to no infectious virus was present in vaccinated marmosets. When marmosets were challenged 35 days post-vaccination, only the marmosets that received the sham inoculation developed viremia as detected by qRT-PCR indicating that the vaccinated monkeys were protected. The samples with the highest viremia as determined by qRT-PCR were evaluated for infectious virus by standard plaque assay and an average of 5.4 log_10_ PFU/mL was detected on day 2 post-challenge.

**Fig 4:**
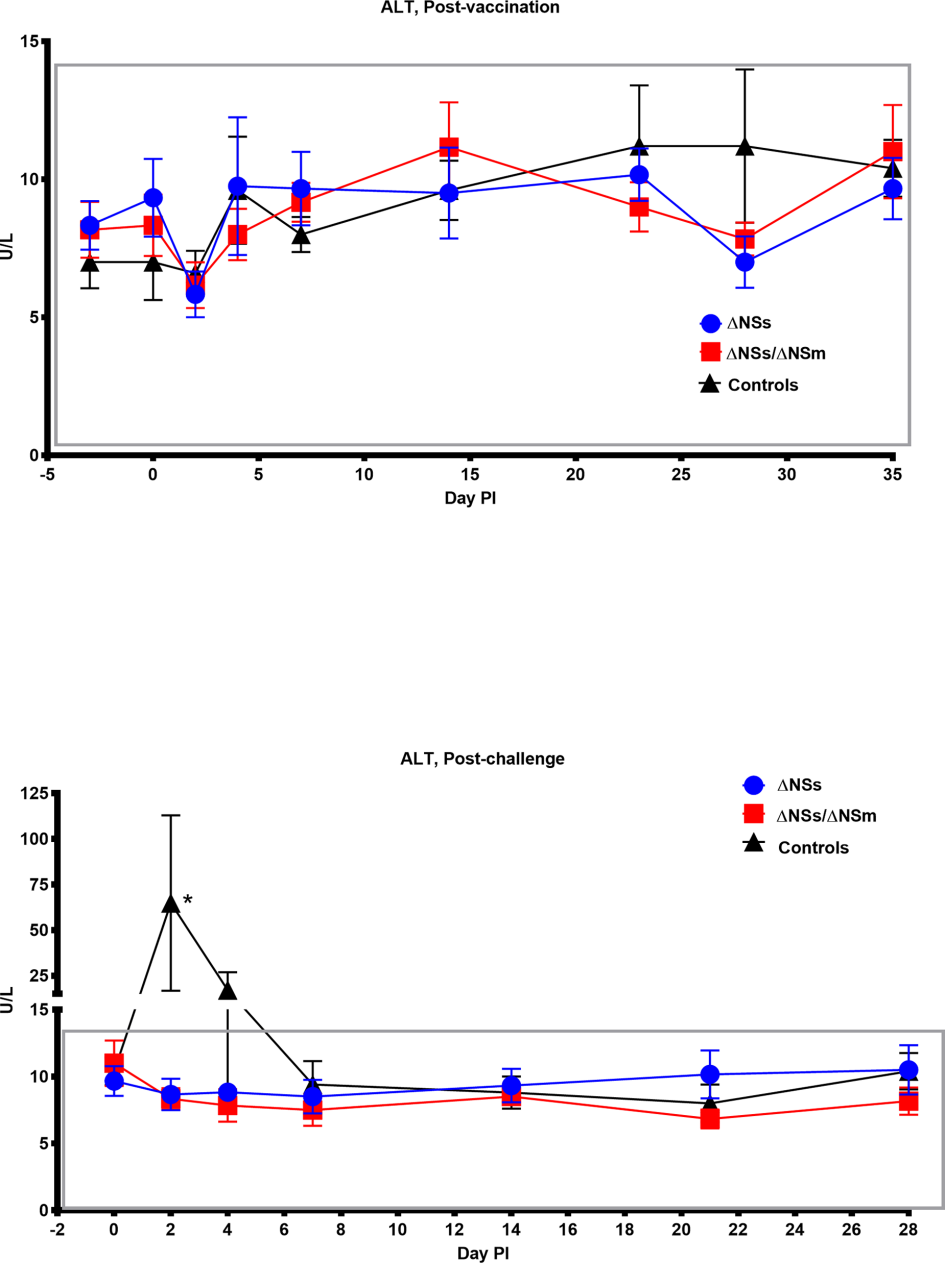
**Alanine aminotransferase (ALT) levels of marmosets post-vaccination (top) and post-challenge (bottom).** ALT levels in the blood of marmosets post-vaccination (top) with rZH501-ΔNSs (n = 6), rZH501-ΔNSs-ΔNSm (n = 6), or sham inoculated controls (n = 5) and post-challenge (bottom) with 6 log_10_ PFU of the virulent strain ZH501. The boxes represent the normal ALT reference range variability in healthy animals. The symbols represent the mean value and the error bars represent the standard error of the mean. Asterisk (*) indicates significantly different values when comparing post-challenge (bottom) ΔNSs vs. controls and ΔNSs-ΔNSm vs. controls p = 0.0002.

In our previous model development study we observed marked aberrations in hematological and chemistry values from marmosets exposed to RVFV. In particular, the liver enzyme ALT was significantly increased when marmosets were exposed to RVFV subcutaneously. We therefore collected blood samples post-vaccination and post-challenge for hematological and chemical analyses. Overall, no significant change in the hematology and clinical chemistry values of the vaccinated animals was observed post-vaccination or post-challenge. For example, the ALT levels (Fig 4) in vaccinated animals were similar post-vaccination and post-challenge. As expected, the control animals did have an increase in ALT levels on day 2 post-challenge which suggest that the vaccinated animals were protected from RVFV-induced liver disease. We expected to see a change in other hematology and chemistry values in the sham inoculated controls post-challenge, but none were significantly different compared to baseline which is in contrast to what we observed in our previous model development study.

All vaccinated animals developed neutralizing antibodies by day 14 post-vaccination (Table 1). These titers peaked by day 21 post-vaccination, which were slightly higher for animals receiving the ΔNSs rRVFV. The RVFV IgG titers peaked on day 35 post-vaccination and similar to the neutralizing antibody titer results, were slightly higher for animals receiving the ΔNSs rRVFV. However, there was no statistically significant difference in the neutralizing antibody response and anti-RVFV total IgG response for animals receiving the ΔNSs rRVFV vs. ΔNSs-ΔNSm rRVFV (Table 2). The only statistically significant antibody responses was observed in animals receiving either the ΔNSs rRVFV or ΔNSs-ΔNSm rRVFV vaccine compared to the sham-vaccinated controls. Overall the adjusted sum_OD_ values were low (including those for RVFV challenged controls), which is most likely due to the secondary antibody being raised against rhesus macaques and not marmosets (a marmoset specific antibody does not exist). The neutralizing antibody and RVFV IgG titers increased for all animals post-challenge.

**Table 1:** Neutralizing antibody titers and anti-RVFV total IgG adjusted Sum_OD_ ELISA results of plasma from vaccinated and control animals.

Inoculum	rZH501-ΔNSs			rZH501-ΔNSs-ΔNSm			Sham Inoculated Controls		
	Mean PRNT_50_ ± STDEV	Mean PRNT_80_ ± STDEV	Anti-RVFV IgG mean Sum_OD_ ± STDEV	Mean PRNT_50_ ± STDEV	Mean PRNT_80_ ± STDEV	Anti-RVFV IgG mean Sum_OD_ ± STDEV	Mean PRNT_50_ ± STDEV	Mean PRNT_80_ ± STDEV	Anti-RVFV IgG mean Sum_OD_ ± STDEV
Day 7 post-vaccination	1:867 ± 393	1:<400 ± 0	0.004 ± 0.006	1:<400 ± 0	1:<400 ± 0	-0.005 ± 0.0477	1:<400 ± 0	1:<400 ± 0	0.008 ± 0.023
Day 14 post-vaccination	1:23,467 ± 14,964	1:3,533 ± 4,709	0.111 ± 0.105	1:10,933 ± 4,572	1:933 ± 547	0.0741 ± 0.045	1:<400 ± 0	1:<400 ± 0	-0.002 ± 0.013
Day 21 post-vaccination	1:59,733 ± 34,976	1:8,267 ± 9,407	0.382 ± 0.125	1:26,667 ± 20,001	1:6,400 ± 3,505	0.258 ± 0.193	1:<400 ± 0	1:<400 ± 0	-0.003 ± 0.007
Day 28 post-vaccination	1:29,867 ± 17,488	1:13,867 ± 6,292	0.689 ± 0.168	1:10,733 ± 8,647	1:3,000 ± 2,814	0.384 ± 0.225	1:<400 ± 0	1:<400 ± 0	0.009 ± 0.004
Day 35 post-vaccination	1:25,707 ± 15,989	1:7,333 ± 9,253	1.052 ± 0.289	1:10,667 ± 8,507	1:1,867 ± 2,286	0.749 ± 0.492	1:<400 ± 0	1:<400 ± 0	0.003 ± 0.023
Day 7 post-challenge	1:56,533 ± 39,331	1:15,200 ± 9,149	1.757 ± 0.459	1:34,133 ± 19,271	1:10,133 ± 4,253	1.553 ± 0.717	1:3,360 ± 5,311	1:800 ± 490	0.001 ± 0.007
Day 14 post-challenge	1:90,667 ± 70,390	1:32,533 ± 21,648	2.011 ± 0.748	1:72,533 ± 65,601	1:16,000 ± 7,838	1.659 ± 0.691	1:71,680 ± 28,043	1: 8,640 ± 5,724	0.378 ± 0.307
Day 21 post-challenge	1:78,933 ± 73,493	1:21,333 ± 17,013	1.482 ± 0.656	1:59,733 ± 71,802	1:17,067 ± 17,013	1.338 ± 0.675	1:184,320 ± 133,513	1:46,080 ± 33,378	1.294 ± 0.594
Day 28 post-challenge	1:36,267 ± 17,013	1: 9,067 ± 4,253	1.867 ± 1.013	1:14,933 ± 5,226	1:4,267 ± 1,652	1.374 ± 0.609	1:307,202 ± 289,635	1:97,280 ± 68,692	1.232 ± 0.417

**Table 2:** Comparison of the neutralizing antibody response and anti-RVFV total IgG response by Tukey’s multiple comparisons test; NS = not significant.

Inoculum	rZH501-ΔNSs vs. rZH501-ΔNSs-ΔNSm			rZH501-ΔNSs vs. Sham Inoculated Controls			rZH501-ΔNSs-ΔNSm vs. Sham Inoculated Controls		
	PRNT_50_	PRNT_80_	ELISA	PRNT_50_	PRNT_80_	ELISA	PRNT_50_	PRNT_80_	ELISA
Day 7 post-vaccination	NS	NS	NS	NS	NS	NS	NS	NS	NS
Day 14 post-vaccination	NS	NS	NS	NS	NS	NS	NS	NS	NS
Day 21 post-vaccination	NS	NS	NS	NS	NS	NS	NS	NS	NS
Day 28 post-vaccination	NS	NS	NS	NS	NS	p = 0.0347	NS	NS	NS
Day 35 post-vaccination	NS	NS	NS	NS	NS	p = 0.0005	NS	NS	p = 0.0180
Day 7 post-challenge	NS	NS	NS	NS	NS	p<0.0001	NS	NS	p<0.0001
Day 14 post-challenge	NS	NS	NS	NS	p = 0.0346	p<0.0001	NS	NS	p<0.0001
Day 21 post-challenge	NS	NS	NS	p = 0.0228	p = 0.0273	NS	p = 0.0055	p = 0.0076	NS
Day 28 post-challenge	NS	NS	NS	p<0.0001	p<0.0001	NS	p<0.0001	p<0.0001	NS

The tissues were tested for viral RNA by qRT-PCR and all tissues from the vaccinated animals were negative except for the spleen and axillary lymph node of one animal receiving the rZH501-ΔNSs vaccine and the skeletal muscle and cerebrum of one animal receiving the rZH501-ΔNSs-ΔNSm vaccine (Table 3). All of the control animals had detectable viral RNA in multiple tissues (primarily the lymphoid tissues). However, these RNA values were low in both the vaccinated and control animals and are most likely insignificant since no tissues were positive by IHC and infectious virus was not detected by cytopathic effect assay. Furthermore, histologic findings directly attributable to RVFV infection were not observed.

**Table 3:** Virus detected in the tissues of marmosets by qRT-PCR or CPE assay at the study endpoint.

Inoculum	Animal #	Tissue	Tissue Titer by qRT-PCR (PFUe/g)	Infectious Virus by CPE Assay
rZH501-ΔNSs	9944	Spleen	2.3	Negative
	9944	Axillary LN	3.3	Negative
	All Animals	Liver	Negative	Negative
		Cerebrum		
		Kidney		
		Lung		
		Heart		
		Adrenal Gland		
		Inguinal LN		
		Mesenteric LN		
		Duodenum		
		Jejunum		
		Ileum		
		Ovaries/Testis		
		Skeletal Muscle		
		Bone Marrow		
		Retina		
rZH501-ΔNSs-ΔNSm	9939	Skeletal Muscle	2.4	Negative
	9909	Cerebrum	4.2	Negative
	All Animals	Spleen	Negative	Negative
		Axillary LN		
		Liver		
		Kidney		
		Lung		
		Heart		
		Adrenal Gland		
		Inguinal LN		
		Mesenteric LN		
		Duodenum		
		Jejunum		
		Ileum		
		Ovaries/Testis		
		Bone Marrow		
		Retina		
Sham Inoculated Controls	9940	Spleen	2.9	Negative
	9940	Axillary LN	2.7	Negative
	9940	Inguinal LN	3.5	Negative
	7516	Spleen	3.0	Negative
	7516	Axillary LN	3.9	Negative
	7516	Inguinal LN	3.1	Negative
	9785	Kidney	2.7	Negative
	9785	Skeletal Muscle	3.1	Negative
	9785	Axillary LN	3.5	Negative
	9785	Inguinal LN	4.0	Negative
	9785	Mesenteric LN	2.9	Negative
	9935	Lung	3.8	Negative
	9935	Duodenum	4.3	Negative
	9935	Jejunum	3.1	Negative
	JK330	Spleen	3.5	Negative
	JK330	Adrenal Gland	2.7	Negative
	JK330	Skeletal Muscle	3.6	Negative
	JK330	Axillary LN	3.6	Negative
	JK330	Inguinal LN	2.9	Negative
	2/5 Animals	Spleen	Negative	Negative
	1/5 Animals	Axillary LN		
	1/5 Animals	Inguinal LN		
	4/5 Animals	Kidney		
	3/5 Animals	Skeletal Muscle		
	4/5 Animals	Mesenteric LN		
	4/5 Animals	Lung		
	4/5 Animals	Duodenum		
	4/5 Animals	Jejunum		
	4/5 Animals	Adrenal Gland		
	All Animals	Liver		
		Cerebrum		
		Heart		
		Ileum		
		Ovaries/Testis		
		Bone Marrow		
		Retina		

In summary, both the ΔNSs and ΔNSs-ΔNSm vaccine candidates were found to be safe and immunogenic in the current study. The vaccinated marmosets exhibited no signs of clinical illness post-vaccination and post-challenge and developed strong neutralizing antibody titers. Additionally, minimal viremia as detected by qRT-PCR was observed post-vaccination and no viral RNA was identified in the serum of vaccinated animals post-challenge.

## Discussion

RVFV significantly impacts livestock and human health making it a good target for a one-health prevention approach through animal vaccination. Livestock vaccination during non-epidemic periods or as an early countermeasure against early outbreaks could eliminate one of the main sources of human infection and limit the scope of epidemics. However, previous RVFV outbreaks are generally recognized only after human cases are diagnosed [49, 50]. Additionally, a human vaccine is still needed to protect veterinarians involved in vaccination programs, slaughterhouse workers and farmers. Human vaccination to protect the general public could be required if efficient spread of RVFV by anthropophilic mosquito species occurs. Finally, since RVFV is a potential agent of bioterrorism, a human vaccine is needed to protect against the threat posed by intentional dissemination.

Currently, there is no fully licensed vaccine for veterinary or human use available in non-endemic countries. In endemic countries, there is no clear guidance for livestock vaccinations to prevent RVF outbreaks. Furthermore, previous veterinary vaccines for RVF has been plagued with numerous concerns such as high manufacturing costs, a poorly defined genetic identity, poor efficacy, no capacity to differentiate vaccinated from naturally infected livestock, and the risk of vaccination in pregnant animals due to associated teratogenesis and abortion [2, 18, 51, 52]. Next-generation veterinary vaccines are being developed that overcome many of these limitations. However, regulatory and economic challenges continue to preclude the development of a human vaccine. Clearly, the licensing of both a veterinary and human vaccine is needed for RVFV. A logical strategy is to use a common approach for veterinary and human vaccine development with the goal to reduce development and licensing costs.

Bird et al. developed rationally designed vaccine candidates based on the complete deletion of two known RVFV virulence factors, the NSs and NSm genes [24, 25]. The rRVFV vaccine candidates containing the insertion of the enhanced green fluorescent protein and the precise deletion of the NSs gene alone (ΔNSs:GFP rRVFV) or the NSs/NSm genes in combination (ΔNSs:GFP-ΔNSm rRVFV) were found to be highly attenuated, immunogenic, and efficacious in the rat lethal disease model [24]. Importantly, a robust antibody response was observed with both vaccine candidates demonstrating that the double-genetic deletions of the entire RVFV NSs and NSm genes does not significantly decrease overall vaccine efficacy compared to the single-genetic deletion of the NSs. The design of a vaccine candidate with attenuating deletions on multiple virus genome segments provides enhanced safety by reducing the possibility of reversion to full virulence via either RVFV polymerase nucleotide substitution or gene segment reassortment with field strains. The insertion of the GFP gene was removed (due to vaccine licensure concerns containing a foreign gene) and the double-genetic deletion rRVFV was further evaluated in a natural RVFV host. This ΔNSs-ΔNSm rRVFV vaccine was demonstrated to be protective and immunogenic in sheep without producing clinical illness in these animals [24, 25]. The vaccine was nonteratogenic in pregnant sheep, which is critical to demonstrate the safety needed for a veterinary vaccine in a natural RVFV host. Additionally, the ΔNSs-ΔNSm rRVFV vaccine was demonstrated to be compatible with a differentiation of infected and vaccinated animals (DIVA) enzyme-linked immunosorbent assay (ELISA) [24, 25, 53]. Here, we expand upon that work and completed an evaluation of the single deletion mutant (ΔNSs rRVFV) and double deletion mutant (ΔNSs-ΔNSm rRVFV) vaccine candidates in marmosets.

We demonstrate that both the ΔNSs rRVFV and ΔNSs-ΔNSm rRVFV vaccine candidates were found to be safe and immunogenic in the current study. The vaccinated marmosets received 5 log_10_ PFU of virus and exhibited no signs of clinical illness, experienced no significant weight loss, or temperature changes post-vaccination and post-challenge. Additionally, minimal viral RNA was observed post-vaccination and no viral RNA was identified in the serum of vaccinated animals post-challenge. No significant change in the hematology and clinical chemistry values of the vaccinated animals was observed post-vaccination or post-challenge. In contrast, the liver enzyme ALT was significantly increased in sham-vaccinated control animals suggesting that the vaccinated animals were protected from RVFV-induced liver disease. Collectively, these results demonstrate the general safety of these vaccine candidates in NHPs. However, more extensive safety testing such as an assessment of neurovirulence would be necessary for advance development efforts. This would be especially important for the ΔNSs vaccine candidate which was shown to cause a uniform fatal encephalitis after intranasal, but not subcutaneous exposure in C57BL/6 mice [54]. A separate study utilizing another recombinant ZH501 RVFV strain lacking the NSs gene demonstrated that CD1 mice can occasionally develop encephalitis (5% mortality was reported) after intraperitoneal exposure [55]. These studies suggest that additional attenuating mutations other than NSs may be important for the safety of RVFV vaccine candidates.

The immunogenicity of the ΔNSs and ΔNSs-ΔNSm rRVFV vaccine candidates was noteworthy. All vaccinated animals developed high neutralizing antibody titers by day 14 post-vaccination, which peaked by day 21 post-vaccination. Antibody titers were slightly higher for animals receiving the ΔNSs rRVFV than animals vaccinated with the double-deletion ΔNSs-ΔNSm rRVFV, but this difference was not found to be statistically significant. The ΔNSs rRVFV may be slightly more immunogenic because of the single deletion in a known RVFV virulence factor compared to ΔNSs-ΔNSm rRVFV, which has two gene deletions. We would expect that the ΔNSs-ΔNSm rRVFV would be more attenuated presumably due to reduced *in vivo* virus replication and less stimulation of the antiviral immune response. However, we did detect similar levels of viral RNA in the blood on day 2 post-vaccination for both the single and double deletion viruses. It is possible that differences in the kinetics or magnitude of virus replication occur between the single and double deletion viruses that we didn’t detect with the current study design. However, even with the slight reduction in antibody titers all animals were completely protected by both vaccine candidates. Since the double-genetic deletions of the entire RVFV NSs and NSm genes does not significantly decrease overall vaccine efficacy, it makes sense to pursue this as the lead candidate for licensure. The ΔNSs-ΔNSm rRVFV is likely safer due to multiple attenuating lesions leading to a reduced possibility of reversion to full virulence.

It is difficult to directly compare antibody titers as an indication of protective immunity to those of previous studies with other RVFV vaccines because of the differences in the candidates/approach, species level differences in immunity, and timing for assessing the response. However, a retrospective study of human volunteers (n = 598) receiving a three-dose regimen (days 0, 7, and 28) of inactivated TSI-GSD-200 vaccine reported that subjects developed a mean PRNT_80_ of 1:237 [17]. The live attenuated MP-12 vaccine was evaluated in rhesus macaques where vaccinated animals demonstrated PRNT_80_ values of ≥1:640 [19, 56]. In the current study, the mean PRNT_80_ ranged from 1:6,400 to 1:8,267 on day 21 post-vaccination, indicating that the level of neutralizing antibody was substantially higher to that demonstrated in earlier studies of RVFV vaccines in NHP models or in human volunteers. However, it is difficult to directly compare antibody titers between various studies for the aforementioned reasons.

The virulent virus challenge dose used in this study (6 log_10_ PFU/mL) was chosen based on our previous model development effort, which indicated that we would likely see 50% mortality with the sham-vaccinated control animals. Surprisingly, no mortality was observed for sham-vaccinated animals, which may be a result of the age of the animals used in the current study which ranged from 1 to 3 years old. The age of the animals used in our previous model development effort were 2 to 11 years old with the majority of the animals being older (between 8–11 years old). In fact, the animals that succumbed to RVFV by subcutaneous exposure were 10–11 years old [38]. Another possible difference in the model development study vs. the current study is the use of different sources for the animals, which may have resulted in different genetic backgrounds of the marmosets. While this is highly speculative, it is possible that genetics plays a role in the susceptibility to RVFV infection in NHPs, which has been demonstrated in the RVF rat model. For example, Peters and Anderson used breeding experiments to demonstrate that a dominant gene determines resistance to fatal RVFV-induced liver disease [57, 58]. Clearly, more studies are needed to further characterize the RVF marmoset model and determine the likelihood for the development of severe disease. Despite the lack of mortality, the sham-vaccinated control animals did become viremic as detected by qRT-PCR and experience an increase in the liver enzyme ALT. In contrast, the vaccinated animals did not experience any adverse reactions and viral RNA was not detected in the serum. A previous study of the live attenuated RVFV vaccine MP-12 in rhesus macaques, which is also a non-lethal NHP model, observed post-vaccination viremia detected by plaque assay in 1/3 of vaccinated monkeys and included a slight increase in the liver enzyme AST [56]. Our results suggest that the complete deletion of the NSs and NSs/NSm genes affords a more attenuated phenotype, but still generates a robust antibody response.

In summary, both the ΔNSs and ΔNSs-ΔNSm vaccine candidates have many desired features for human vaccine development. No vaccine-induced adverse reactions, signs of clinical illness or infectious virus were detected in the vaccinated marmosets. The vaccinated animals received a single dose of vaccine that led to the development of robust neutralizing antibody titers that provided complete protection against viremia and liver disease. Our results provide the basis for further development of the ΔNSs and ΔNSs-ΔNSm rRVFV as safe and effective human RVFV vaccines for this significant public health threat.

## Supporting information

S1 Table Summary of the individual animals age, sex, and weight. (DOCX)

S1 Fig **Individual weights of marmosets post-vaccination (top) and post-challenge (bottom).** Percent change in baseline of weights of marmosets post-vaccination (top) with rZH501-ΔNSs (n = 6), rZH501-ΔNSs-ΔNSm (n = 6), or sham inoculated controls (n = 5) and post-challenge (bottom) with 6 log_10_ PFU of the virulent strain ZH501. The symbols represent the mean value and the error bars represent the standard error of the mean.
(TIF)

S2 Fig **Individual temperatures of marmosets post-vaccination (top) and post-challenge (bottom).** Percent change in baseline of the temperature of marmosets post-vaccination (top) with rZH501-ΔNSs (n = 6), rZH501-ΔNSs-ΔNSm (n = 6), or sham inoculated controls (n = 5) and post-challenge (bottom) with 6 log_10_ PFU of the virulent strain ZH501. The symbols represent the mean value and the error bars represent the standard error of the mean.
(TIF)

S3 Fig **Individual viremia determined by qRT-PCR in marmosets post-vaccination (top) and post-challenge (bottom).** RNA detected by qRT-PCR in marmosets post-vaccination (top) with rZH501-ΔNSs (n = 6), rZH501-ΔNSs-ΔNSm (n = 6), or sham inoculated controls (n = 5) and post-challenge (bottom) with 6 log_10_ PFU of the virulent strain ZH501. The symbols represent the mean value and the error bars represent the standard error of the mean. Because of the difficulty viewing the results on day 2 PI, the animal ID’s with RNA detected are in bold text in the legend. The dashed line represents the assay LOD. PFUe, plaque-forming unit equivalent.
(TIF)
