# Supplementary material for: Attenuation and efficacy of live-attenuated Rift Valley fever virus vaccine candidates in non-human primates
Source: PLoS Negl Trop Dis. 2018 May 9;12(5):e0006474. doi: 10.1371/journal.pntd.0006474 (PMC5962102; doi:10.1371/journal.pntd.0006474)
Supplement: S1 Table — (DOCX) [file pntd.0006474.s001.docx]

| **Animal ID** | **DOB** | **Inoculum** | **Age when Infected** | **Sex** | **Initial Weight(g)** |
| --- | --- | --- | --- | --- | --- |
| M9914 | Aug-09 | **rZH501-∆NSs** | 2 | F | 310.00 |
| M9868 | Mar-09 | **rZH501-∆NSs** | 2 | F | 330.00 |
| M9920 | Dec-08 | **rZH501-∆NSs** | 3 | F | 380.00 |
| M9944 | Feb-10 | **rZH501-∆NSs** | 1 | F | 270.00 |
| M9864 | Dec-09 | **rZH501-∆NSs** | 2 | M | 270.00 |
| M7373 | Dec-09 | **rZH501-∆NSs** | 2 | F | 290.00 |
| M7371 | Mar-09 | **rZH501-∆NSs-∆NSm** | 2 | F | 360.00 |
| M9939 | Dec-09 | **rZH501-∆NSs-∆NSm** | 2 | F | 270.00 |
| M9909 | Jun-07 | **rZH501-∆NSs-∆NSm** | 4 | F | 380.00 |
| M7556 | Oct-05 | **rZH501-∆NSs-∆NSm** | 6 | M | 380.00 |
| M7372 | Mar-09 | **rZH501-∆NSs-∆NSm** | 2 | F | 330.00 |
| M9910 | Feb-09 | **rZH501-∆NSs-∆NSm** | 2 | F | 350.00 |
| M9940 | Feb-10 | **Sham Inoculated Control** | 1 | F | 260.00 |
| M7516 | Aug-05 | **Sham Inoculated Control** | 6 | M | 330.00 |
| M9785 | Oct-04 | **Sham Inoculated Control** | 7 | M | 400.00 |
| M9935 | Jan-10 | **Sham Inoculated Control** | 1 | M | 350.00 |
| JK330 | Jul-08 | **Sham Inoculated Control** | 3 | F | 292.00 |
