# Supplementary figures and images for: Attenuation and efficacy of live-attenuated Rift Valley fever virus vaccine candidates in non-human primates
Source: PLoS Negl Trop Dis. 2018 May 9;12(5):e0006474. doi: 10.1371/journal.pntd.0006474 (PMC5962102; doi:10.1371/journal.pntd.0006474)

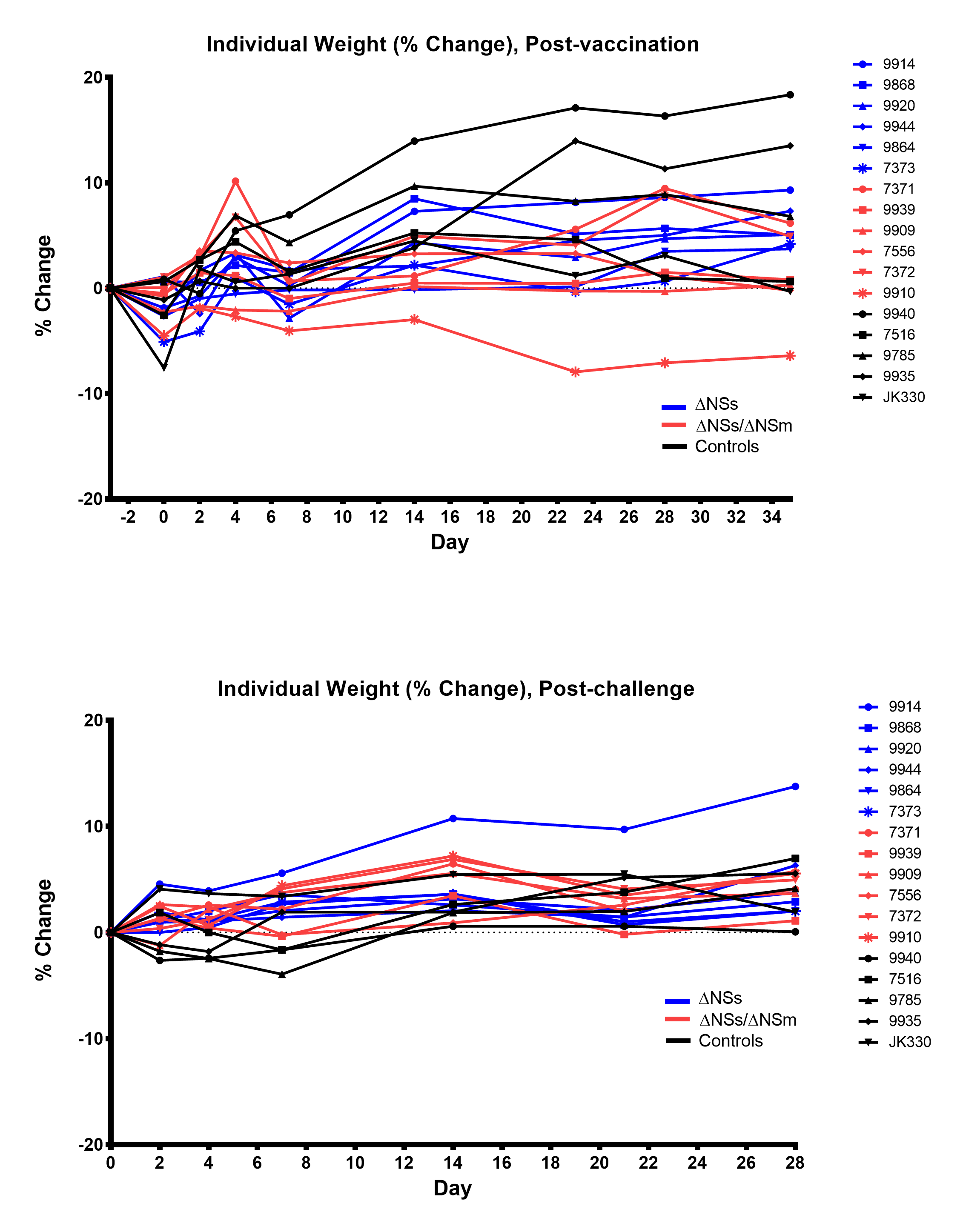

Supplement: S1 Fig — Individual weights of marmosets post-vaccination (top) and post-challenge (bottom). Percent change in baseline of weights of marmosets post-vaccination (top) with rZH501-ΔNSs (n = 6), rZH501-ΔNSs-ΔNSm (n = 6), or sham inoculated controls (n = 5) and post-challenge (bottom) with 6 log10 PFU of the virulent strain ZH501. The symbols represent the mean value and the error bars represent the standard error of the mean. (TIF) [file pntd.0006474.s002.tif]

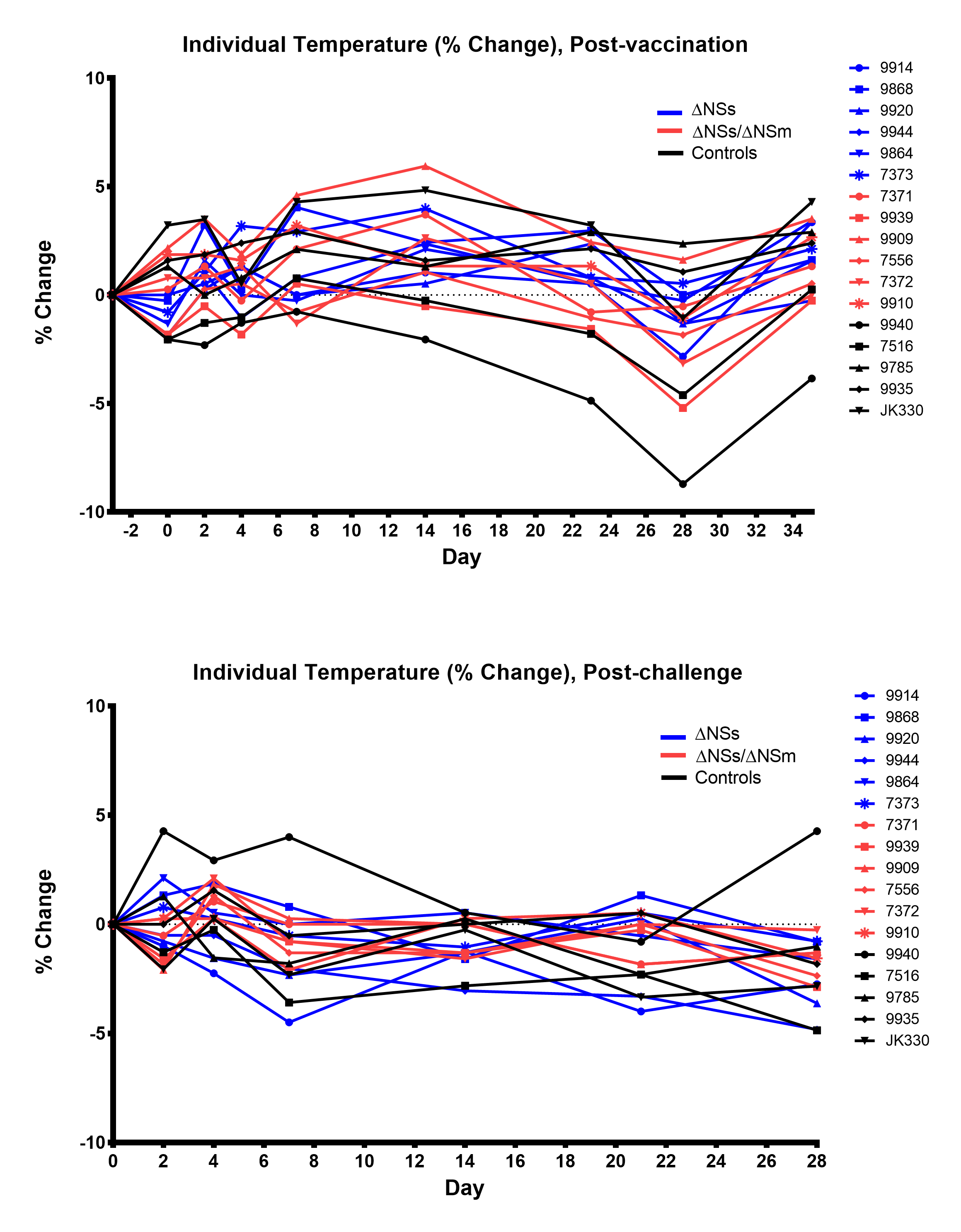

Supplement: S2 Fig — Individual temperatures of marmosets post-vaccination (top) and post-challenge (bottom). Percent change in baseline of the temperature of marmosets post-vaccination (top) with rZH501-ΔNSs (n = 6), rZH501-ΔNSs-ΔNSm (n = 6), or sham inoculated controls (n = 5) and post-challenge (bottom) with 6 log10 PFU of the virulent strain ZH501. The symbols represent the mean value and the error bars represent the standard error of the mean. (TIF) [file pntd.0006474.s003.tif]

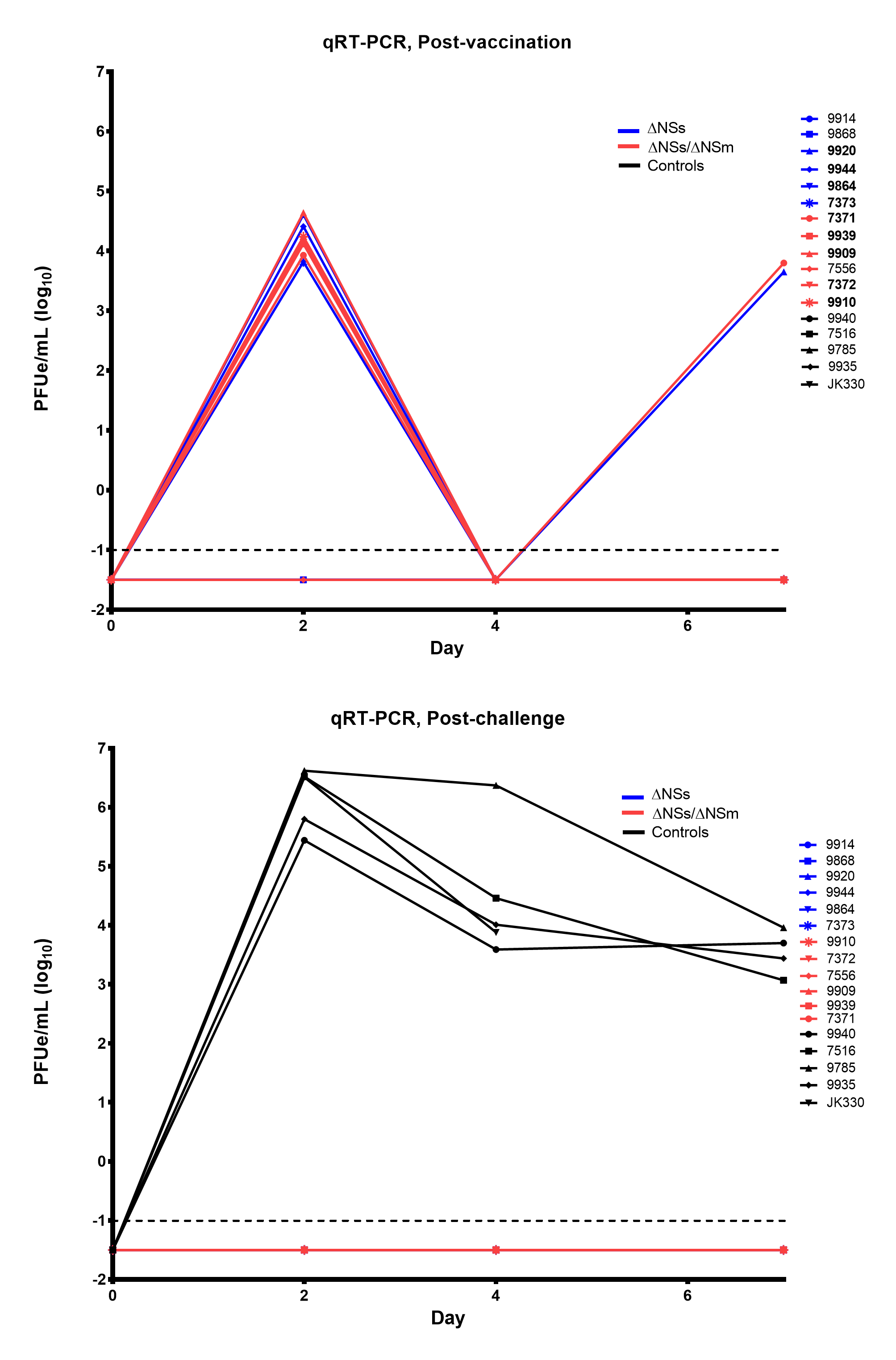

Supplement: S3 Fig — Individual viremia determined by qRT-PCR in marmosets post-vaccination (top) and post-challenge (bottom). RNA detected by qRT-PCR in marmosets post-vaccination (top) with rZH501-ΔNSs (n = 6), rZH501-ΔNSs-ΔNSm (n = 6), or sham inoculated controls (n = 5) and post-challenge (bottom) with 6 log10 PFU of the virulent strain ZH501. The symbols represent the mean value and the error bars represent the standard error of the mean. Because of the difficulty viewing the results on day 2 PI, the animal ID’s with RNA detected are in bold text in the legend. The dashed line represents the assay LOD. PFUe, plaque-forming unit equivalent. (TIF) [file pntd.0006474.s004.tif]
